# A commentary on “The role of coagulopathy and subdural hematoma thickness at admission in predicting the prognoses of patients with severe traumatic brain injury: a multicenter retrospective cohort study from China”

**DOI:** 10.1097/JS9.0000000000003420

**Published:** 2025-09-08

**Authors:** Ling Chen, Hongjie Chen

**Affiliations:** a Department of Science and Education, 900th Hospital, Fuzhou, Fujian Province, China; b Department of Neurosurgery, 900th Hospital, Fuzhou, Fujian Province, China

*Dear Editor,*

Severe traumatic brain injury (TBI) remains one of the most devastating neurological emergencies globally, with complex pathophysiological changes and high rates of mortality and disability^[1,2]^. In the recently published article by Chen et al., the authors proposed and validated a novel prognostic model – X1 – that integrates coagulopathy and subdural hematoma (SDH) thickness at admission to predict the 6-month functional outcome of patients with severe TBI. This multicenter retrospective cohort study contributes meaningfully to the ongoing effort of improving individualized prognostication in neurotrauma. This article has been performed in accordance with the TITAN 2025 guideline[3].

The novelty of the X1 index lies in its simple yet clinically meaningful classification of patients into four strata, based on the presence or absence of coagulopathy and SDH thickness threshold of 14.05 mm. Compared with classical indicators and models, including CRASH and IMPACT, the X1 model demonstrated superior predictive accuracy, with AUCs exceeding 0.9 and consistently strong C-index values in both training and validation cohorts. Importantly, the authors utilized machine learning algorithms to rank feature importance, further highlighting the robustness of X1 as a prognostic biomarker.

One of the key strengths of this study is its practical clinical relevance. Coagulopathy is highly prevalent in severe TBI patients and contributes significantly to progressive hemorrhagic injury and poor neurological outcomes^[4,5]^. Integrating coagulopathy with SDH thickness – an easily obtainable imaging parameter – into a composite index for early risk stratification is both intuitive and impactful. Moreover, the nomogram and decision curve analysis presented by the authors offer clinicians a user-friendly tool to guide early therapeutic decisions and communication with patients’ families.

Nevertheless, as the authors acknowledged, the current study relied on conventional coagulation markers such as PLT, INR, and APTT. These laboratory parameters reflect only a limited aspect of the complex coagulation cascade and do not capture the dynamic interaction between clot formation, stabilization, and fibrinolysis. In this context, thromboelastography (TEG) emerges as a promising tool. TEG provides a comprehensive, real-time evaluation of clot kinetics, strength, and lysis, and has been increasingly adopted in neurocritical care to guide transfusion and hemostatic therapy[6]. Future studies may benefit from incorporating TEG-derived parameters into prognostic models like X1, potentially improving their predictive power and guiding targeted interventions more effectively.

Additionally, while the current study demonstrated excellent internal and external validation across three institutions in China, further prospective multicenter studies – particularly across diverse geographic and ethnic populations – are warranted to enhance generalizability.

In conclusion, Chen et al. have introduced a pragmatic and accurate prognostic tool that holds promise for improving early outcome prediction in severe TBI. The integration of advanced coagulation profiling techniques, such as thromboelastography, in future model iterations may further enhance the precision and clinical utility of such tools.

## Data Availability

This commentary draws solely upon publicly accessible, previously published sources.
